# Cytomegalovirus reactivation with high viral load in a patient of coronavirus disease 2019 acute respiratory distress syndrome: a case report

**DOI:** 10.1186/s13256-023-03819-y

**Published:** 2023-05-16

**Authors:** Sourav Pal, Atul Garg, Anupam Agarwal, Ujjala Ghoshal, Pooja Singh, Jitendra S. Chahar, Mohan Gurjar

**Affiliations:** 1 Department of Microbiology, Sanjay Gandhi Post Graduate Institute of Medical Sciences, Lucknow, 226014 India; 2 Critical Care Medicine, Sanjay Gandhi Post Graduate Institute of Medical Sciences, Lucknow, 226014 India

**Keywords:** CMV, Reactivation, RT-PCR, COVID-19, Ganciclovir

## Abstract

**Introduction:**

Cytomegalovirus establishes life-long latency after primary infection in childhood. Cytomegalovirus reactivation has been well reported in immune-compromised patients; however, in the last few years it has been observed that cytomegalovirus reactivation also occurs in critically ill patients without exogenous immunosuppression, which increases length of intensive care unit stay and mortality rate.

**Case report:**

A 63-year-old Indian male, without any known comorbidity, developed severe coronavirus disease 2019 and was admitted to the intensive care unit. He received remdesivir, tocilizumab, steroids, anticoagulants, and empiric antibiotics over the next 3 weeks. However, his clinical condition did not improve much, and during the 9th week of illness his condition started deteriorating and routine bacterial cultures, fungal cultures, and cytomegalovirus real-time polymerase chain reaction on blood were negative. His clinical condition worsened rapidly, which led to the need for invasive mechanical ventilation. Tracheal aspirate bacterial and fungal culture showed no growth, but cytomegalovirus real-time polymerase chain reaction showed 21,86,000 copies/mL in tracheal aspirates. After 4 weeks of ganciclovir treatment, the patient improved clinically and was discharged. Currently he is doing well and able to do his routine activity without the need of oxygen.

**Conclusion:**

Timely management with ganciclovir is associated with favorable outcome in cytomegalovirus infection. Thus, it can be suggested that treatment should be initiated with ganciclovir if a patient with coronavirus disease 2019 has high cytomegalovirus load in tracheal aspirates, along with unexplained and prolonged clinical and/or radiological features.

## Introduction

Approximately 60–80% of immunocompetent adults are cytomegalovirus (CMV) seropositive worldwide. Following primary infection, CMV remains latent in monocytes and macrophages in multiple organs. Due to altered immune status, CMV reactivation has been well reported in immunocompromised patients such as transplant recipients, patients undergoing chemotherapy or immunomodulatory treatment, and patients with chronic immunodeficiency. However, in the last few years it has been observed that CMV reactivation also occurs in critically ill patients without exogenous immunosuppression, which increases length of intensive care unit (ICU) stay and mortality rate [1].

Severe coronavirus disease 2019 (COVID-19) caused by severe acute respiratory syndrome coronavirus 2 (SARS CoV-2) is characterized by pneumonia, lymphopenia, exhausted T-cell response, and a cytokine storm with high levels of interleukin (IL)-6, tumor necrosis factor (TNF)-α, monocyte chemoattractant protein (MCP)-1, macrophage inflammatory protein (MIP)-1α, and so on. There is massive infiltration of macrophages into the lungs, and many of these macrophages carry latent CMV that may be reactivated by SARS-CoV-2 itself, toll-like receptor stimulation, and cytokines like IL-1, IL-6, TNF-α, and granulocyte-macrophage colony-stimulating factor (GM-CSF) [2]. All these factors might contribute to reactivation of CMV in critically ill patients with COVID-19. Here we present an interesting case of CMV reactivation and associated complications in a critically ill patient with COVID-19.

## Case report

A 63-year-old male of Indian ethnicity, nonsmoker, nonalcoholic, vegetarian, self-employed professional, without any coexisting disease, developed fever and dry cough for 7 days and mild difficulty in breathing for 1 day before he visited his local general practitioner. He lived alone as his family was visiting their relatives in another city. He was empirically started on oral azithromycin 500 mg and cetirizine 10 mg once daily along with Paracetamol 650 mg twice daily, and was advised to undergo a real-time polymerase chain reaction (RT-PCR) testing for SARS CoV-2 and sputum bacterial culture.

RT-PCR for SARS CoV-2 was positive, and in view of rapidly progressing breathing difficulty, he was admitted to ICU facility of our COVID-19 hospital. At time of hospital admission patient was conscious, oriented, with normal cough power, and adequate swallowing. Upper and lower limb power was around 4/5. His consciousness level was normal initially but after sometime he started becoming drowsy, his respiratory rate was 34–36 breaths per minute and with SpO_2_ of 84% on room air; he was hemodynamically stable [blood pressure (BP) 136/82, heart rate of 114 beats per minute]. Laboratory parameters such as hemogram, renal function test, liver function test, and procalcitonin were within normal limits, certain parameters were deranged like C-reactive protein (CRP) 45 ng/ml, serum ferritin 611 ng/ml, D dimer 2.8 µg/ml, and plasma fibrinogen level 575 mg/dl. Chest X-ray showed bilateral patchy infiltrates.

He required continuous non-invasive ventilation (NIV) for moderate acute respiratory distress syndrome (ARDS), and received intravenous remdesivir 100 mg for 10 days and tocilizumab 400 mg once. Later on, he received intravenous methyl prednisolone 60 mg twice daily for 5 days and then continued as once daily. He also received enoxaparin 60 mg subcutaneously (s/c) twice daily, with other supportive care over next 3 weeks. During 2nd week, empiric antibiotic cefoperazone sulbactam 1.5 mg twice daily was started. However, his clinical condition did not improve much, and was shifted to non-COVID-19 ICU for further management once he became negative for SARS CoV-2 in RT-PCR.

At admission to non-COVID-19 ICU (5th week of illness, W-5), he continued to require NIV for hypoxemia. His hemogram, bilirubin, serum glutamic oxaloacetic transaminase (SGOT), and serum glutamic pyruvic transferase (SGPT) were within normal limits. His urea and creatinine were mildly elevated, and he had dyselectrolytemia (sodium 132 mEq/l, potasium 3.3 mmol/l). Blood and urine cultures were negative; he had high HbA1c 8.3% (diabetes of recent onset) with severity score, sequential organ failure assessment (SOFA) score of 5, and acute physiology and chronic health evaluation (APACHE) II score of 10. The high-resolution computed tomography (HRCT) of the chest showed extensive lung parenchyma involvement with features of fibrosis (bilaterally). Over the next 3 weeks, steroids were gradually tapered, antibiotics were deescalated from cefoperazone–sulbactam to clarithromycin and later stopped. Initially, patient was put on intermittent ventilation by NIV with alternate non-rebreather mask (NRBM), and later on his oxygenation was maintained on venturi mask at 0.3 FiO_2_. Throughout his stay, the patient was on anticoagulant medication and received daltaperin 5000 IU s/c once daily.

**Fig. 1:**
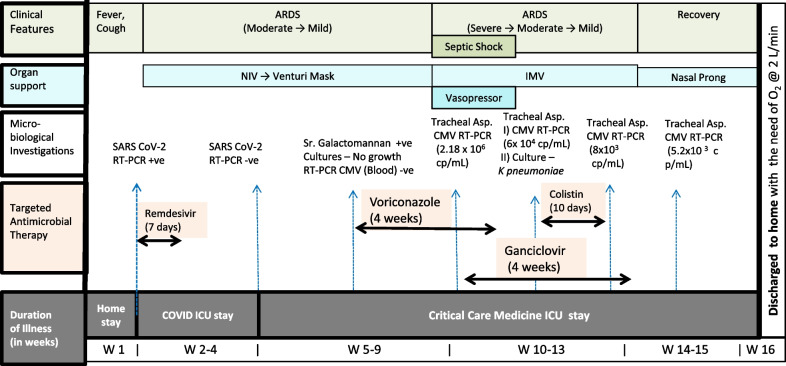
Time line of clinical disease and management in patient. *ARDS* acute respiratory distress syndrome, *NIV* noninvasive ventilation, *MV* invasive mechanical ventilation

During the 9th week (W-9) of illness his condition (hypoxemia) started deteriorating. He became tachypnoeic with retention of CO_2_ for which he received intermittent NIV again with increased FiO_2_ up to 0.6%; however, he became drowsy, with PaCO_2_ rising up to 108 mmHg, for which he was intubated and invasive mechanical ventilation initiated. Repeat bacterial cultures, fungal cultures, and CMV RT-PCR on blood were negative, but serum galactomannan was positive (1.3 ODI units) and intravenous voriconazole (4 mg/kg twice daily) with empiric intravenous antibiotic cefoperazone-sulbactum (2 gm twice daily) was started. Over the next week (W-10), his clinical condition rapidly worsened as he developed circulatory shock, and required vasopressors and hydrocortisone 200 mg/day. His oxygenation worsened, with increased oxygen requirement and multiple cycles of prone ventilation performed. Antimicrobials escalated (meropenem and teicoplanin). Computed tomography pulmonary angiography (CTPA) was done to rule out pulmonary thromboembolic condition. Cardiac event was also ruled out. Serum galactomannan decreased to 0.7 ODI units, tracheal aspirate bacterial and fungal culture showed no growth, coagulase negative staphylococcus was isolated in one blood culture bottle, and cytomegalovirus RT-PCR showed 21,86,000 copies/ml in tracheal aspirates (Fig. 1).

**Fig. 2:**
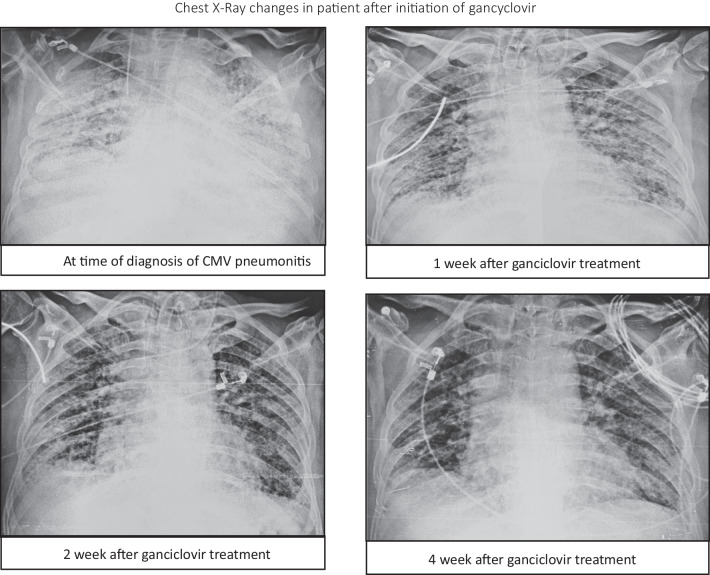
Chest X-ray changes in patient after initiation of ganciclovir

The patient was CMV Immunoglobulin G (IgG) antibody positive and an infectious disease specialist diagnosed the case as CMV reactivation, and suggested intravenous injection of ganciclovir 5 mg/kg/day. Over the next 3–4 days, he recovered from circulatory shock. After 2 weeks of ganciclovir treatment, the patient improved clinically and repeat CMV RTPCR in tracheal aspirate showed 60,900 copies/ml (Fig. 1). However, at the time of weaning, he again had increased tracheal secretion with new infiltrate on his chest X-ray. Repeat tracheal culture showed growth of extremely drug resistant *Klebsiella pneumoniae* sensitive only to colistin, so colistin (4 million IU 8 hourly) was administered for 10 days. Ganciclovir was continued for a total of 4 weeks with gradual reduction of CMV copies in tracheal aspirates and clearing of chest X-ray.(Fig. 2). His clinical condition improved, and he was successfully weaned from mechanical ventilation (4-week duration). Subsequently, after 15th week of the illness (W-15), patient got discharged home on 2 l per minute oxygen through nasal prongs, valgenciclovir 450 mg tablet orally twice daily, and levofloxacin 750 mg tablet orally once daily for 5 days. Patient was required to take prednisolone 5 mg tablet once daily at 10 a.m. for 5 days, then 2.5 mg once daily for 5 days, and then 2.5 mg every alternate day for a final 5 days. In further follow-up after 12 months of discharge, he was doing well and able to do his routine activity without the need for oxygen.

## Discussion

Our case briefly describes successful management of a prolonged clinical course of a critically ill patient with COVID-19, who developed CMV reactivation. There was very high CMV viral load in tracheal aspirates and initially he was treated by standard 2-week course of ganciclovir; however, as his viral load in tracheal aspirate was found to be 60,900 copy/mL after 2 weeks of treatment, the ganciclovir treatment was extended for 2 more weeks, and then showed clinical improvement. Finally, he was discharged from the ICU in stable condition. Recently a case series of five patients has been reported from Lisbon, Portugal [3]. All patients were admitted to ICU due to SARS CoV-2 pneumonia and presented with concomitant CMV infection/reactivation during ICU stay, with a median viral load of 516 IU/ml in bronchoalveolar lavage (BAL) specimen.

There are two monocentric studies on CMV reactivation in patients with COVID-19. The first study is from France, including 38 patients with COVID-19 on mechanical ventilation in ICU; 30% of them showed evidence of CMV reactivation at a median of 9 days [interquartile range (IQR) 5–14 days]. The second study of 34 patients reported CMV reactivation at a median of 9 days in 15% of patients with COVID-19 in the ICU, with a median viral load of 4930 IU/mL in both these studies. CMV reactivation was associated with increased duration of mechanical ventilation and prolonged hospital stay [4, 5].

The diagnosis of CMV infection can be easily missed in the ICU on the basis of clinical and radiological findings, as it cannot be differentiated from other respiratory infections and clinical microbiologist and intensivists are not well aware of this emerging infection. Therefore, in critically ill patients with COVID-19, especially those on prolonged mechanical ventilation, steroid therapy, or other immunomodulatory therapy, clinicians should consider CMV pneumonitis in the differential diagnosis whenever patients have unexplained worsening and respiratory failure, and should send respiratory samples for quantitative CMV RT-PCR.

CMV infection and CMV disease are not synonymous terms, and not all patients with infection develop overt clinical disease. As per the third international consensus guidelines on the Management of Cytomegalovirus in Solid-organ Transplantation (2018), CMV viral loads cannot be directly compared across different centers as they are specimen (whole blood/plasma/tissue) and assay specific (Cobas/Artus/others), and it is recommended that each center should establish its own cut off threshold to differentiate between innocent CMV reactivation and CMV disease and manage accordingly; furthermore, the trend of changing viral load is more important than single viral loads. In a recent study involving a lung transplant recipient, quantitative CMV RT-PCR was performed on BAL samples, and it was observed that patients with CMV pneumonia had higher viral loads compared with those without pneumonia and the optimal cut off viral load suggested was 4545 international units/ml [6]. Similarly, in a study on CMV reactivation in patients with COVID-19, median viral load observed was 4930 IU/ml. Apart from the viral parameters, other clinical and laboratory parameters like leukopenia, atypical lymphocytosis, fever, and deranged liver function test (elevated SGOT/SGPT/ bilirubin) should be considered while evaluating CMV reactivation in patients with COVID-19.

## Conclusion

Currently, there is no consensus on the intervention with respect to CMV reactivation in critically ill patients; however, it has been well documented that timely management with ganciclovir 5 mg/kg/day is associated with favorable outcomes in CMV infection. Thus, based on available literature, it can be suggested that treatment should be initiated with ganciclovir if the patient with COVID-19 has high CMV load in tracheal aspirates, along with unexplained and prolong clinical and/or radiological features.

## Data Availability

All relevant data generated during this study are included in this article. Further enquiries can be directed to the corresponding author for Additional data.
